# Pain responses following resistance exercise with and without blood flow restriction in individuals with multiple sclerosis

**DOI:** 10.1007/s00421-025-06052-1

**Published:** 2025-12-19

**Authors:** Christopher E. Proppe, Sean M. Lubiak, Paola M. Rivera, Mason A. Howard, Jeffrey T. Schmidt, Ethan C. Hill

**Affiliations:** 1 Department of Human Performance Studies, Wichita State University, 1845 Fairmount St, Wichita, KS 67260 USA; 2 School of Kinesiology and Rehabilitation Sciences, University of Central Florida, Orlando, FL USA; 3 School of Sport Science, Endicott College, Beverly, MA USA; 4 AdventHealth, Sports Med and Rehab, Orlando, FL USA; 5 College of Medicine, University of Central Florida, 6850 Lake Nona Blvd, Orlando, FL USA

**Keywords:** Exercise induced hypoalgesia, Blood flow restriction, Pain

## Abstract

**Purpose:**

Pain is a common symptom among individuals living with multiple sclerosis (MS). Exercise-induced hypoalgesia (EIH), a reduction in pain sensitivity following resistance exercise, typically requires high loads. Blood flow restriction (BFR) training may offer a lower-load alternative to elicit EIH. This investigation aimed to assess EIH responses following resistance exercise with and without BFR in individuals with MS.

**Methods:**

15 participants diagnosed with MS completed three exercise sessions consisting of isotonic, unilateral leg extensions with the current exercise recommendation (high-load, 70% of 1RM), low-load BFR (LL + BFR, 30% of 1RM with BFR), or low-loads (30% of 1RM without BFR). Pain pressure threshold (“uncomfortable”) and tolerance (“painful”) of the rectus femoris were assessed before and after each exercise session. After each exercise set, participants rated their pain (0–10) which was used for mediation analyses.

**Results:**

Participants completed similar exercise volume (high-load = 3056.0 ± 728.2 kg; low-load = 3208.3 ±  1041.5 kg) but pain pressure threshold was greater (*p* = 0.012–0.025) following LL + BFR (1.04 ± 0.97 Δkgf) compared to high-load (0.37 ± 0.70 Δkgf) and low-load exercise (0.28 ± 0.69 Δkgf). Pain pressure tolerance was greater (*p* = 0.015) following LL + BFR (1.41 ± 1.01 Δkgf) compared to low-load exercise (0.23 ± 1.69 Δkgf). Peak pain was greater (*p* = 0.004–0.030) during LL + BFR (4.3 ± 2.9 au) compared to high-load (1.6 ± 1.7 au) and low-load (2.3 ± 2.4 au) exercise but was not a significant mediator of the EIH response.

**Conclusion:**

LL + BFR elicited a local EIH response compared to the current exercise recommendation (high-load) and low-load resistance exercise. These findings suggest that LL + BFR may be an potent exercise intervention to acutely alter pain sensitivity in patients with MS.

## Introduction

Multiple sclerosis (MS) is a chronic autoimmune disease associated with demyelination of the central nervous system (Wootla et al. 2012). Symptoms of MS include sensory impairment, pain, extremity weakness, decreased physical function, fatigue, and spasticity (Kister et al. 2013). Although each case of MS presents with a unique clinical profile, interventions typically focus on limiting progression of the disease and managing symptoms using a combination of disease-modifying therapies, pharmacological treatment, exercise, lifestyle modifications, and emotional support (Hauser and Cree 2020; McGinley et al. 2021).

While pharmacological therapies and treatments have been partially effective at reducing relapse rates and slowing the progression of MS, they do not address common symptoms such as decreased physical function, which can lead to decreased quality of life and increased risk of comorbidities (Ewanchuk et al. 2018; Dalgas et al. 2019; Hauser and Cree 2020; McGinley et al. 2021). Exercise has been shown to reduce or reverse some symptoms associated with MS including weakness, physical function, and fatigue (Dalgas et al. 2008, 2019). Additionally, an acute reduction in pain sensitivity has been observed after exercise (exercise-induced hypoalgesia [EIH]) (Koltyn 2002; Koltyn et al. 2014). Using exercise to manage pain is of particular relevance in individuals living with MS because pain is a commonly reported symptom (29–91% of patients) (Solaro et al. 2013). EIH can be achieved through multiple modes of exercise but regardless of exercise mode, high intensity and/or prolonged exercise is typically required (Koltyn 2002; Rice et al. 2019). While no single mechanism has been definitively identified, high intensity and/or prolonged exercise may activate one or several proposed mechanisms, including activation of the endogenous opioid system, cannabinoid system, conditioned pain modulation (“pain inhibits pain”), elevated blood pressure (baroreceptor-related mechanisms), descending inhibitory pathways in the central nervous system, and reductions in inflammation (Hughes and Patterson 2019; Vaegter and Jones 2020).

The current exercise recommendation for resistance training for individuals living with MS is 2–3 resistance training sessions per week consisting of 5–10 exercises, 1–3 sets for each exercise, and 8–15 repetitions per set at 60–80% of one repetition maximum (1RM) (Halabchi et al. 2017; Kim et al. 2019). However, not all individuals living with MS may be able to complete resistance exercises with these loads. Alternatively, low-load (30% of 1RM) resistance training with blood flow restriction (LL + BFR) is an alternative to traditional forms of exercise. LL + BFR has been shown to elicit positive muscular adaptations, including increased muscular strength and hypertrophy, in healthy and clinical populations (Lixandrão et al. 2015; Hughes et al. 2017; Grønfeldt et al. 2020). Additionally, LL + BFR has been shown to elicit an acute EIH response similar to high-load resistance training in healthy populations (Hughes and Patterson 2020; Hammert et al. 2024). The positive muscular adaptation, with a lower load, and potential reductions in pain sensitivity may make LL + BFR a useful exercise modality for individuals living with MS.

There is limited evidence examining the acute EIH responses following high-load resistance exercise and LL + BFR in individuals living with MS. Quantifying changes in pain sensitivity after resistance exercise may provide useful information related to EIH response in individuals living with MS. Therefore, the purpose of the present investigation was to assess the EIH responses following the current exercise recommendation for resistance training (high-load), LL + BFR, and low-load resistance exercise in individuals living with MS.

## Methods

For this investigation, 15 participants (Table 1) who had been diagnosed with relapsing-remitting MS were recruited and completed all aspects of this study. An *a priori* power analysis was completed using G*Power (Faul et al. 2007) to determine the appropriate sample size. Alpha was set to 0.05, power to 80%, and reported effect sizes from studies that have examined EIH following BFR exercise (*d* = 0.46 [Hughes and Patterson 2020] and *d* = 0.78 [Hughes et al. 2021]) were used to estimate a necessary sample size of 15 to 18 for the present study design. Due to high levels of variability among individuals living with MS a slightly higher sample size was recruited to minimize the risk of violating normality. Of the 15 participants, 14 (3 men and 11 women) of the participants’ data have been used previously as part of a larger multi-independent variable study for purposes unrelated to the present study. The inclusion criteria were as follows: Expanded Disability Status Score between 0 and 6.5, ability to walk 10 m with or without assistance, no MS relapses during the 30 days preceding enrollment in the investigation, not currently undergoing a rehabilitation protocol, and free of cardiovascular, pulmonary, metabolic, muscular, and/or other medical conditions that would limit the capacity to exercise. Additionally, participants reported no changes in medication usage during the study. This investigation was approved by the University Institutional Review Board for Human Subjects, was in accordance with the ethical standards of the Declaration of Helsinki 2013, participants provided written informed consent before participating in the investigation, and the results are reported in accordance with the CONSORT (Consolidated Standards of Reporting Trials) guidelines (Schulz et al. 2010).

**Table 1:** Participant demographic information

	*n*	Age (y)	Height (cm)	Mass (kg)	BPI Severity	BPI Impact	EDSS	Years since diagnosis
Females	12	40.9 ± 10.8	167.8 ± 10.2	85.4 ± 19.9	1.7 ± 1.4	0.7 ± 1.0	4.0 ± 1.3	9.1 ± 6.7
Males	3	37.3 ± 5.7	184.3 ± 6.3	96.6 ± 17.7	3.4 ± 3.2	2.9 ± 4.0	3.8 ± 1.5	6.0 ± 2.0
Total	15	39.8 ± 9.3	173.5 ± 10.5	86.2 ± 20.4	2.1 ± 1.9	1.1 ± 2.0	4.0 ± 1.3	8.5 ± 6.1

*BPI* Brief Pain Inventory (Short Form)

### Protocol

This investigation utilized a randomized, within-subject crossover design. The intervention order was determined using the randomization function within Excel. Participants completed four visits: one consent and baseline testing visit, followed by three exercise visits with at least 48-hours between visits. Visits were completed in a randomized, counterbalanced order to minimize any potential training effects related to the exercise sessions. During exercise visits, participants completed unilateral leg extensions with a low-load (30% of 1RM), LL + BFR (30% of 1RM, arterial occlusion pressure [AOP] 60%), or a high-load (70% of 1RM). To assess EIH responses, pain pressure threshold and tolerance of the rectus femoris were assessed before and after each exercise intervention. All assessments were completed in a climate-controlled (22℃, ~ 55% humidity) laboratory space.

During the first visit, participants completed the informed consent form, medical history, and the Brief Pain Inventory (Short Form) (Table 1). Participants were then familiarized with the pain pressure threshold and tolerance device (Wagner FPX, Greenwich, CT, USA) and testing procedure, and then completed a one-repetition maximum testing protocol. Prior to strength testing, participants completed a 5-minute self-paced warm-up on a cycle ergometer. Participants were then seated on an isokinetic dynamometer (Biodex System 3, Biodex Medical Systems, Inc. Shirley, NY, USA), and the seat was adjusted such that the axis of rotation of the knee was aligned with the axis of rotation with the dynamometer. The seat position was recorded and used for all subsequent visits. With the dynamometer set to isotonic mode, participants completed a one-repetition maximum protocol to determine the maximal leg extension strength of the dominant leg (Miller 2012). The first set involved 5–10 repetitions at 50% of the estimated 1RM. During subsequent sets, participants completed 2–3 sets of 3–5 repetitions with progressively heavier loads, followed by sets of 1–3 repetitions until the participant could no longer complete a leg extension muscle action through a full range of motion (Miller 2012). Full range of motion was defined as moving from 90° of flexion to full extension of the leg (180°). The maximal load lifted was used to determine working loads during subsequent visits.

### Exercise interventions

Participants completed three exercise intervention visits in a randomized counter-balanced order. Prior to each exercise intervention, participants completed a self-paced 5-minute warm-up on a cycle ergometer. Participants were then seated on and secured to the dynamometer which was set to isotonic mode. During each exercise visit, participants completed isotonic concentric-eccentric unilateral leg extension muscle actions. During the low-load exercise visit, participants completed 75 total repetitions (1 × 30, 3 × 15, 30 s of intraset rest) of leg extension muscle actions at 30% of 1RM with no BFR. During the LL + BFR visit, participants completed 75 total repetitions (1 × 30, 3 × 15, 30 s of intraset rest) of leg extension muscle actions at 30% of 1RM with blood flow restriction (60% AOP). During the high-load exercise visit, participants completed the current exercise recommendation of 3 × 12 (60 s of intraset rest) of leg extension muscle actions at 70% of 1RM without BFR (Halabchi et al. 2017; Kim et al. 2019). After each set of exercise, participants were asked to rate their overall pain during the previous set on a visual analog scale ranging from 0 to 10 (arbitrary units) with three anchor points (zero corresponded to “no pain”, five “moderate pain”, and ten “worst pain”). To determine peak pain during exercise, the highest value reported during each exercise intervention, regardless of which set it occurred in, was identified and used for subsequent analyses. Peak pain was selected based on its relevance to conditioned pain modulation paradigms, which use a nociceptive stimulus to elicit an analgesic response (Lewis et al. 2012).

### Blood flow restriction

Arterial occlusion pressure of the exercising leg was determined at the start of the LL + BFR visit. Participants rested in the laboratory for 10-minutes and then laid in the supine position on a padded table. A 10 × 61–96.5 cm single-chambered nylon cuff (Suji Sub Inc., Orlando, FL USA) was applied to the proximal most portion of the exercising leg. The cuff was connected to a rapid inflating pneumatic device (Hokanson Rapid Cuff Inflation System; Hokanson Inc., Belleview, WA, USA) and progressively inflated while blood flow through the posterior tibial artery was visually monitored using Doppler ultrasound (GE Logiqe, General Electric Medical Systems, Milwaukee, WI, USA). Total occlusion pressure was defined as the lowest pressure required to completely occlude blood flow through the posterior tibial artery. During LL + BFR visits, the BFR cuff was applied in the same position and inflated to 60% of AOP before the first repetition of the first set and deflated following the last repetition of the last set of exercise.

### Pain pressure threshold and tolerance

Mechanical pain sensitivity was assessed at the rectus femoris (halfway between the anterior superior iliac spine and superior aspect of the patella) before and immediately after each exercise session. Assessments were completed unilaterally on the leg that completed the exercise, distal to the region where the BFR cuff was applied. A pressure algometer (Wagner FPX, Greenwich, CT, USA) with a 1 cm^2^ flat rubber tip was applied perpendicular to the target muscle at approximately 1 kg/cm^2^/s. Participants were asked to identify when the pressure became “uncomfortable” and “unbearable” to assess pain pressure threshold and pain pressure tolerance, respectively (Fischer 1987; Andersen et al. 2006). Three measurements were obtained, and the average of the three trials was used for further analysis. To account for individual variations in pain perception and potential inter-day variability, pain pressure threshold and tolerance responses were analyzed as changes from pre-exercise values (post-exercise average–pre-exercise average).

### Statistical analysis

To assess differences in the volume of exercise between the exercise interventions, the total volume load (sets × repetition × load) between the low-load conditions (low-load without BFR and LL + BFR) and the high-load exercise was analyzed using a paired sample *t* test and Hedge’s *g* effect size was calculated. The load-load and LL + BFR exercise interventions were not separated for this analysis because the same set × repetition scheme and load were used during both visits, therefore, the volume was identical. Separate one-way ANOVAs were used to compare pre-exercise pain pressure threshold and tolerance values to ensure consistency across intervention days. Additionally, separate one-way ANOVAs were used to compare changes from pre-exercise values for peak pain during exercise, pain pressure threshold, and pain pressure tolerance. Partial eta-squared effect sizes ($\eta_{\rm{p}}^{2}$) were calculated for each ANOVA, and post-hoc comparisons were performed using Bonferroni correction. An alpha of *p* ≤ 0.05 was considered statistically significant for all comparisons.

**Fig. 1:**
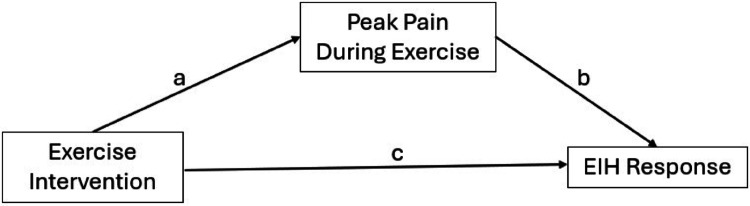
Model of the mediation analysis examining potential effects of peak pain during exercise on the exercise induced hypoalgesia response (EIH). a The effect of the independent variable on the mediator. b The effect of the mediator on the dependent variable. c The direct effect of the independent variable on the dependent variable

Mediation analyses were performed using the SPSS macro PROCESS (Model 4) to evaluate the relationship between exercise intervention and changes in pain pressure threshold and tolerance while using pain during exercise as a mediator (Fig. 1) (Hayes and Little 2022). Peak pain (VAS) during exercise was used as the mediating variable because it has been suggested that pain or discomfort during exercise may be an underlying mechanism of EIH (Hughes and Patterson 2019). The regression coefficient for the indirect effect (path a*b) and direct effect (path c) are presented in unstandardized form and coefficients were considered statistically significant if the confidence intervals did not cross zero (Hayes and Little 2022). The coefficient of determination for the overall model was also calculated. All statistical analyses were performed using IBM SPSS v. 29 (IBM, Armonk, NY, USA).

## Results

### Exercise volume

There was no significant (*p* = 0.501, Hedge’s *g*= − 0.169) difference in total volume load between the high-load (3056.0 ± 728.2 kg) and low-load (3208.3 ± 1041.5 kg) exercise interventions.

### Pain pressure threshold

**Fig. 2:**
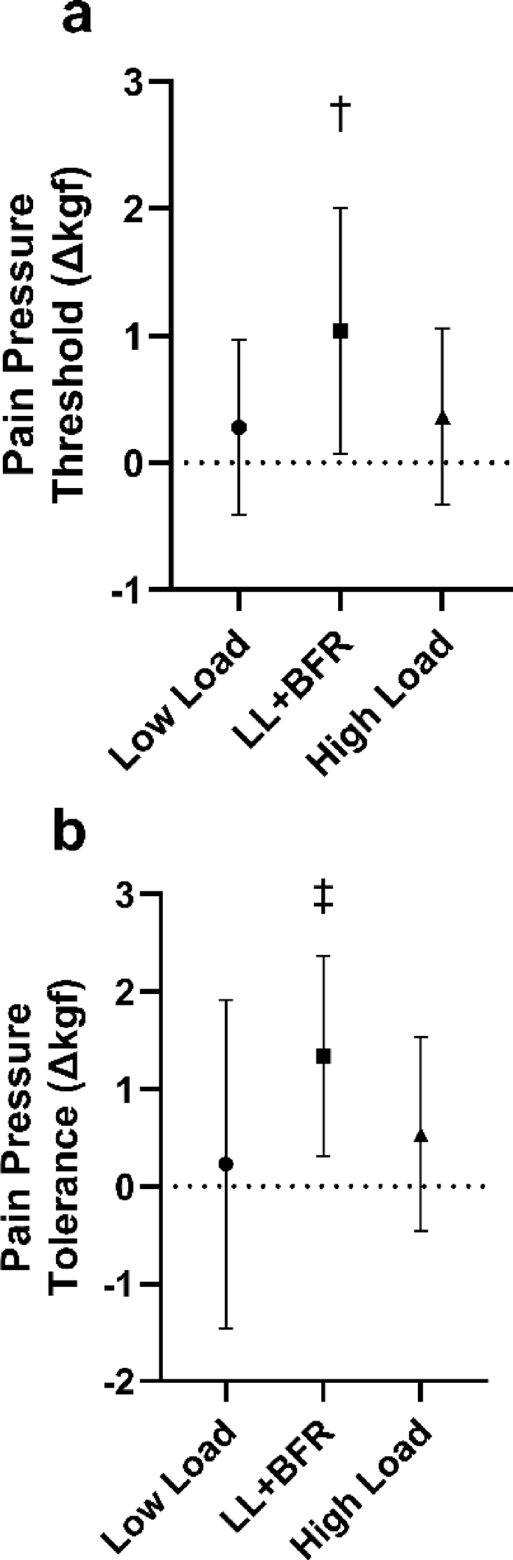
Mean and standard deviation change from pre-exercise values in pressure (Δkgf) for pain pressure threshold (**a**) and tolerance (**b**) of the rectus femoris. Circles indicate low load resistance exercise, squares that low load resistance exercise with blood flow restriction (LL + BFR), and triangles indicate high load resistance exercise. ^†^Signifies that LL + BFR was significantly greater than high load and low load resistance exercise. ^‡^Signifies that LL + BFR was significantly greater low load resistance exercise. *p* ≤ 0.05

There was no significant (*p* = 0.484, $\eta_{\rm{p}}^{2}$ = 0.34) difference in pre-exercise pain pressure threshold values between exercise interventions. There was a significant (*p* = 0.023, $\eta_{\rm{p}}^{2}$ = 0.164) difference in pain pressure threshold between exercise interventions. Follow-up analysis indicated that change in pain pressure threshold was significantly (*p* = 0.012–0.025) greater following the LL + BFR (1.04 ± 0.97 Δkgf) exercise compared to the high-load (0.37 ± 0.70 Δkgf) and low-load exercise (0.28 ± 0.69 Δkgf). There was no significant (*p* = 0.765) difference between the high-load and low-load exercise (Fig. 2a).

### Pain pressure tolerance

There was no significant (*p* = 0.900, $\eta_{\rm{p}}^{2}$ = 0.005) difference in pre-exercise pain pressure tolerance values between exercise interventions. There was a significant (*p* = 0.041, $\eta_{\rm{p}}^{2}$ = 0.141) difference in pain pressure tolerance between exercise interventions. Follow-up analysis indicated that change in pain pressure tolerance was significantly (*p* = 0.015) greater following the LL + BFR (1.41 ± 1.01 Δkgf) exercise compared to the low-load exercise (0.23 ± 1.69 Δkgf). There was no significant (*p* = 0.067) difference between the LL + BFR and high-load (0.73 ± 1.34 Δkgf) exercise or the high-load and low-load exercise (*p* = 0.518) (Fig. 2b).

### Peak pain during exercise

**Fig. 3:**
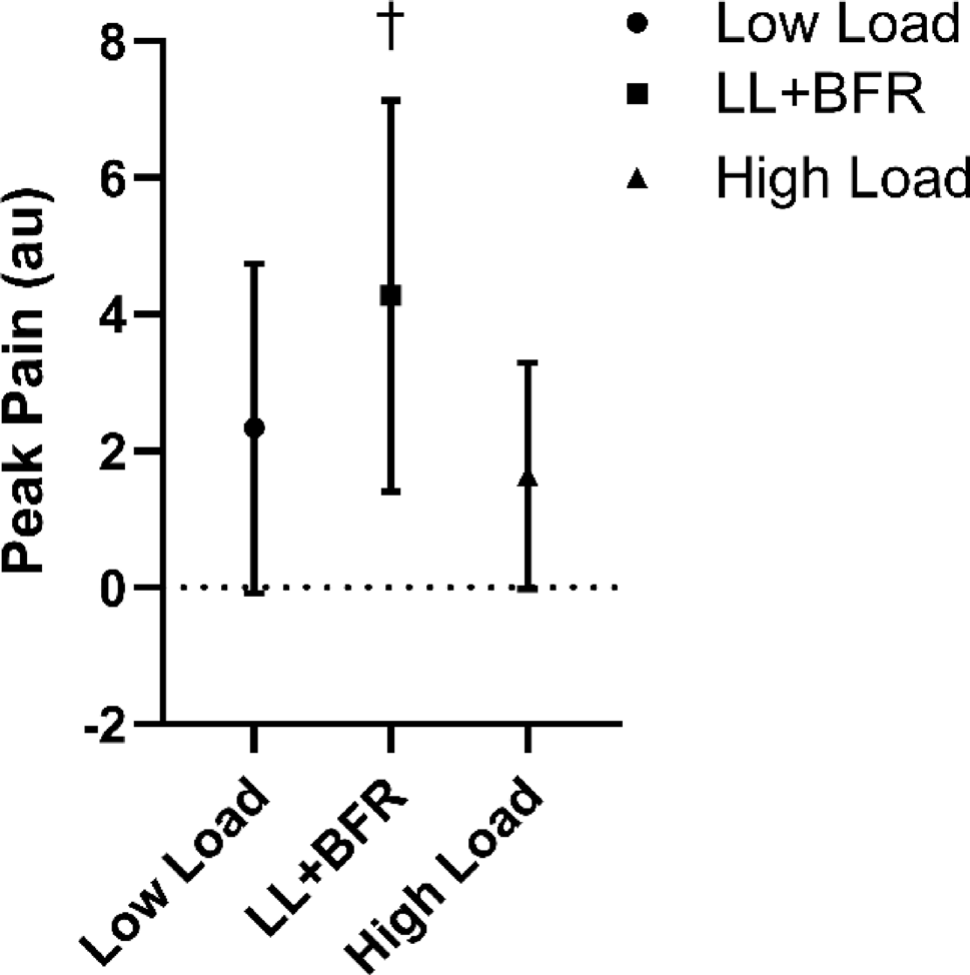
Mean and standard deviation of the maximal perceived pain (arbitrary units). Circles indicate low load resistance exercise, squares that low load resistance exercise with blood flow restriction (LL + BFR), and triangles indicate high load resistance exercise. ^†^Signifies that LL + BFR was significantly greater than high load and low load resistance exercise. *p* ≤ 0.05

There was a significant (*p* = 0.011, $\eta_{\rm{p}}^{2}$ = 0.192) difference in peak pain during exercise between interventions. Follow-up analysis indicated that peak pain during exercise was significantly (*p* = 0.004–0.030) higher during the LL + BFR (4.3 ± 2.9 au) exercise compared to the high-load (1.6 ± 1.7 au) and low-load (2.3 ± 2.4 au) exercise. There was no significant (*p* = 0.422) difference between the high-load and low-load exercise (Fig. 3).

### Mediation analysis

For peak pain during exercise, the confidence intervals for the estimate of the coefficient of the indirect effect of peak pain during exercise on pain pressure threshold and pain pressure tolerance both contain zero, indicating that peak pain during exercise did not significantly mediate the relationship between exercise (LL + BFR and high-load) intervention and pain pressure threshold or tolerance (Table 2).

**Table 2:** Coefficients, bootstrap confidence intervals, and significance statistics for mediation analyses

Pain pressure threshold			
Exercise intervention	Total effect	Direct (c)	Indirect (a + b)
*LL: constant*			
LL + BFR	0.761 (0.1759, 1.3461)*	0.8598 (0.1874, 1.5322)*	− 0.0988 (− 0.6217, 0.3999)
HL	0.0872 (− 0.4979, 0.6723)	0.1175 (− 0.5137, 0.7487)	− 0.0302 (− 0.3170, 0.1791)
Overall analyses r^2^	0.1642		
*Pain pressure tolerance*			
Exercise intervention	Total	Direct (c)	Indirect (a + b)
*LL: constant*			
LL + BFR	1.1771 (0.2385, 2.1157)*	1.0870 (0.0408, 2.1332)*	0.0857 (− 0.3135, 0.5655)
HL	0.3032 (− 0.6354, 1.2418)	0.3780 (− 0.7082, 1.4643)	− 0.0614 (− 0.4229, 0.3646)
Overall analyses r^2^	0.1412		

*p < 0.05. LL: low-load, LL + BFR: low-load with blood flow restriction, HL: high-load

## Discussion

The purpose of this investigation was to examine the EIH response following LL + BFR, the current exercise recommendation (high-load), and low-load resistance exercise in individuals diagnosed with relapsing-remitting MS. The results of the present investigation indicated that, in general, pain pressure threshold was significantly higher following LL + BFR (1.04 ± 0.97 Δkgf) compared to the high-load (0.37 ± 0.70 Δkgf) and low-load exercise (0.28 ± 0.69 Δkgf) despite similar exercise volumes between the low-load exercises (LL + BFR and low-load; 3,208.3 ± 1,041.5 kg) and the high-load exercise (3,056.0 ± 728.2 kg). Furthermore, pain pressure tolerance was significantly greater following LL + BFR (1.41 ± 1.01 Δkgf) compared to the low-load exercise (0.23 ± 1.69 Δkgf). While peak pain during exercise was significantly greater during LL + BFR (4.3 ± 2.9 au) exercise compared to the high-load (1.6 ± 1.7 au) and low-load (2.3 ± 2.4 au) exercise, the mediation analyses indicated that peak pain was not a significant mediator of the observed EIH responses.

The results of the present investigation are partially consistent with previous investigations (Hughes and Patterson 2020; Karanasios et al. 2022) that have quantified the EIH response following LL + BFR in healthy populations. For example, in healthy males and females, pain pressure threshold of the biceps brachii increased similarly following unilateral elbow flexion exercise with LL + BFR (4.83 ± 1.9 kg/cm^2^ to 6.44 ± 1.9 kg/cm^2^; 1 × 30, 3 × 15 at 30% of 1RM with 40%-50% AOP) and high-load resistance exercise (4.87 ± 2.07 kg/cm^2^ to 5.83 ± 1.9 kg/cm^2^; 4 × 10 at 70% of 1RM) (Karanasios et al. 2022). Furthermore, in recreationally active men and women, pain pressure threshold increased at the quadriceps similarly following unilateral leg press exercise with LL + BFR (3.72 ± 2.24 Δkgf; 1 × 30, 3 × 15 at 30% of 1RM, 80% AOP) and high-load resistance exercise (1.68 ± 1.53; 4 × 10 at 70% of 1RM) (Hughes and Patterson 2020). However, in the present investigation, pain pressure threshold was significantly greater following LL + BFR exercise compared to high-load exercise. It has been suggested that EIH responses for non-BFR exercise may be related to exercise intensity, volume, and/or duration (Koltyn 2002; Koltyn et al. 2014). Furthermore, it has been shown that completing non-BFR unilateral leg extension exercise (50% of 1RM) to high levels of fatigue (8 out of 10) resulted in a significant increase in pain pressure threshold of the rectus femoris compared to quiet rest (Hogge et al. 2025). Conversely, the low fatigue condition (3 out of 10) resulted in no significant change in pain pressure threshold of the rectus femoris (Hogge et al. 2025). Although total volume load was similar in the present investigation, it is possible that the fatigue associated with wearing BFR cuffs (Loenneke et al. 2013; Hill et al. 2022) and accumulation of metabolic byproducts (Millet and Lepers 2004; Sugaya et al. 2011; Suga et al. 2012; Franz et al. 2020) associated with occluded venous blood flow may have resulted in a greater EIH response following LL + BFR compared to LL.

The currently available evidence supports the effectiveness of exercise in eliciting EIH in the healthy population but there is limited evidence related to EIH in individuals diagnosed with relapsing-remitting MS. Acute exercise, in general, has been shown to elicit EIH responses in chronic pain populations, although there is variability among investigations and chronic pain conditions (Rice et al. 2019). Pain is a common symptom associated with MS, with 29%–91% of patients reporting varying levels of pain (Solaro et al. 2013). Chronic exercise has been shown to reduce pain (Demaneuf et al. 2019), decrease fatigue (Sá 2014), and improve quality of life in individuals with relapsing-remitting MS (Motl and Gosney 2008). However, to our knowledge, no other investigation has examined the acute EIH response (as determined by mechanical pain assessments) following LL + BFR and the current exercise recommendation. The results of the present investigation suggest that LL + BFR is an effective exercise mode to elicit an EIH response among individuals with relapsing-remitting MS. The results of the present investigation, and the evidence suggesting that LL + BFR is well tolerated and induces positive musculoskeletal adaptions among individuals living with relapsing-remitting MS (Freitas et al. 2021; Mañago et al. 2024a; Hill et al. 2024) suggest that LL + BFR may be a useful multifunctional exercise intervention.

The underlying mechanisms that mediate EIH following exercise are not fully understood but several possible mechanisms have been proposed (Koltyn et al. 2014; Hughes and Patterson 2019; Vaegter and Jones 2020). One potential mechanism is the concept that “pain inhibits pain” (i.e. diffuse noxious inhibitory control or conditioned pain modulation) (Le Bars et al. 1992). When a noxious stimulus is detected, there is an activation descending pain modulatory mechanisms that inhibits the detection or decreases the perceived intensity of subsequent noxious stimuli (Le Bars et al. 1992; Le Bars and Willer 2008; van Wijk and Veldhuijzen 2010; Ramaswamy and Wodehouse 2021). In the present investigation, participants reported higher levels of peak pain during the LL + BFR (4.3 ± 2.9 au) exercise compared to the high-load (1.6 ± 1.7 au) and low-load (2.3 ± 2.4 au) exercise. These results are consistent with a recent review which found LL + BFR discomfort during exercise than when exercise is completed without BFR (Spitz et al. 2022).

However, the mediation analysis in the present investigation suggested that peak pain during exercise was not related to the EIH outcomes. This is supported by the finding that the total and direct pathways were significant (Table 2), while the indirect effect was not, indicating that LL + BFR influences EIH primarily through a direct pathway rather than through peak pain as a mediator. These results are partially consistent with previous investigations (Hughes and Patterson 2020; Hammert et al. 2024). Specifically, Hughes and Patterson (2020) reported that muscle discomfort during exercise mediated 58% of the total effect of LL + BFR on changes in pain pressure threshold while Hammert et al. (2024) found no evidence that peak discomfort ratings during LL + BFR had a mediating effect of pain pressure threshold (Hughes and Patterson 2020; Hammert et al. 2024). The results of the present investigation and those of Hammert et al. (2024) suggest an alternative mechanism may be a larger mediator of the EIH following LL + BFR compared to perceived pain during exercise (Hammert et al. 2024).

Several other physiological responses related to exercise have also been proposed as potential mechanisms that facilitate EIH, including elevated blood pressure and/or baroreceptor stimulation during exercise, increased metabolic byproducts in the working muscle (muscle metaboreflex), and early recruitment and recruitment and fatigue of higher-order motor units (Sugaya et al. 2011; Bell et al. 2018; Domingos and Polito 2018; Fatela et al. 2019; Hughes and Patterson 2019; Singer et al. 2020; Franz et al. 2020). In the present study, LL + BFR elicited a greater EIH response than the low-load and high-load interventions, although the exercise volume was the same across the interventions. In non-occluded conditions, EIH is typically related to the intensity and duration of exercise (Vaegter and Jones 2020), with higher loads and/or longer durations being required to induce EIH. Given the similar exercise volume between the interventions, it is possible that the use of the BFR cuff in the LL + BFR intervention provided a stronger stimulus during exercise, resulting in greater activation of one or a combination of these mechanisms compared to the stimulus provided by the low-load and high-load interactions.

## Conclusion

In the present investigation, LL + BFR elicited a strong local EIH response that was greater than the current exercise recommendation (high-load) and low-load resistance exercise. Specifically, pain pressure threshold was significantly greater following LL + BFR compared to the current exercise recommendation and low-load exercise. Additionally, pain pressure tolerance was significantly greater following LL + BFR compared to low-load exercise. Although perceived pain during exercise was significantly greater during LL + BFR, it was not a significant mediator of the EIH response. Although LL + BFR elicited higher peak pain scores, discomfort during exercise is typically a transient event that subsides after removal of the BFR cuffs. Additionally, previous investigations have shown that this mode of exercise is well tolerated among individuals living with MS (Mañago et al. 2024b; Swink et al. 2025; Schmidt et al. 2025). Although further investigation is needed to elucidate the mechanisms associated with EIH following LL + BFR, these findings suggest that LL + BFR may be a potent mode of exercise to alter acute pain sensitivity in individuals living with relapsing-remitting MS.

## Data Availability

The datasets generated and/or analyzed during the current study are available from the corresponding author on reasonable request.

## Abbreviations

1RM: One repetition maximum
ANOVA: Analysis of variance
au: Arbitrary units
BFR: Blood flow restriction
CONSORT: Consolidated standards of reporting trials
EIH: Exercise-induced hypoalgesia
kgf: Kilogram-force
LL: Low-load
MS: Multiple sclerosis
